# Bactericidal Activity of Eugenol-Rich *Syzygium aromaticum* Essential Oil Against Multidrug-Resistant Bacteria Isolated from Commercial Eggs

**DOI:** 10.3390/pathogens15090910

**Published:** 2026-08-29

**Authors:** Rosa Mareyli Gayosso-San Juan, Nallely Rivero-Perez, María Inés Nicolás-Vázquez, Rosa Isabel Higuera-Piedrahita, Lenin Rangel-López, Benjamín Valladares-Carranza, Jorge Alberto Cortes-Morales, José Esteban Aparicio-Burgos, Deyanira Ojeda-Ramírez, Adrian Zaragoza-Bastida

**Affiliations:** 1 Instituto de Ciencias Agropecuarias, Universidad Autónoma del Estado de Hidalgo, Avenida Universidad 133, Colonia San Miguel Huatengo, Santiago Tulantepec de Lugo Guerrero C.P. 43775, Hidalgo, Mexico; ga280669@uaeh.edu.mx (R.M.G.-S.J.); dojeda@uaeh.edu.mx (D.O.-R.); 2 Departamento de Ciencias Químicas, Facultad de Estudios Superiores Cuautitlán Campo 1, Universidad Nacional Autónoma de México, Cuautitlán Izcalli C.P. 54714, Estado de México, Mexico; nicovain@yahoo.com.mx; 3 Laboratorio 3, Unidad de Investigación Multidisciplinaria, Facultad de Estudios Superiores Cuautitlán, Carr. Cuautitlán-Teoloyucan Km 2.5, Cuautitlán Izcalli C.P. 54714, Estado de México, Mexico; rhiguera05@comunidad.unam.mx; 4 División Académica en Ciencias Agropecuarias, Universidad Juárez Autónoma de Tabasco, Carretera Villahermosa-Teapa Kilómetro 25+2 Ranchería la Huasteca 2da Sección, Villahermosa C.P. 86298, Tabasco, Mexico; ralolenin@gmail.com; 5 Centro de Investigación y Estudios Avanzados en Salud Animal, Facultad de Medicina Veterinaria y Zootecnia, Universidad Autónoma del Estado de México, km 15.5 Carretera Panamericana Toluca-Atlacomulco, Toluca C.P. 50200, Estado de Mexico, Mexico; bvalladaresc@uaemex.mx; 6 Escuela de Estudios Superiores del Jicarero, Universidad Autónoma del Estado de Morelos, Carretera Galeana-Tequesquitengo s/n, Colonia El Jicarero, Jojutla C.P. 62909, Morelos, Mexico; ing_cortesmorales@yahoo.com.mx; 7 Escuela Superior de Apan, Universidad Autónoma del Estado de Hidalgo, Carretera Apan-Calpulalpan, Km 8, Chimalpa Tlalayote, Apan C.P. 43920, Hidalgo, Mexico; jose_aparicio@uaeh.edu.mx

**Keywords:** eugenol, pathogens, resistance

## Abstract

The occurrence of multidrug-resistant (MDR) bacteria in foods of animal origin is a public health concern that underscores the need to investigate natural antimicrobial alternatives. Although *Syzygium aromaticum* essential oil exhibits antibacterial activity, evidence regarding its bactericidal effects against MDR bacteria recovered from commercial eggs remains limited. This study evaluated the bactericidal activity of eugenol-rich *S. aromaticum* essential oil (SAEO) and explored its potential antibacterial mechanisms. SAEO was obtained by steam distillation and chemically characterized by gas chromatography–mass spectrometry (GC–MS). Its antibacterial activity was evaluated by determining the minimum inhibitory concentration (MIC), minimum bactericidal concentration (MBC), bacterial growth kinetics, and changes in membrane permeability. Molecular docking was performed to examine the interactions of eugenol with dihydropteroate synthase (DHPS) and β-ketoacyl-acyl carrier protein synthase III (FabH). Eugenol was the predominant constituent (73.9%), followed by dihydroeugenyl acetate (14.1%) and β-caryophyllene (6.0%). SAEO exhibited bactericidal activity against all tested strains, with MIC and MBC values ranging from 0.125 to 0.25 mg/mL and from 0.25 to 0.50 mg/mL, respectively. SAEO also produced sustained bacterial growth inhibition and concentration-associated changes in membrane permeability. Molecular docking predicted favorable interactions of eugenol with DHPS and FabH; however, these interactions require experimental validation. Overall, the findings demonstrate the in vitro bactericidal activity of eugenol-rich SAEO against MDR bacteria recovered from commercial eggs and support further investigation of its potential application in food-safety interventions.

## 1. Introduction

Antimicrobial resistance (AMR) is recognized as one of the greatest threats to global public health because it compromises the effectiveness of therapies used to prevent and treat infectious diseases in both humans and animals [1]. In 2019, bacterial AMR was directly responsible for an estimated 1.27 million deaths and contributed to approximately 4.95 million deaths worldwide [1]. The emergence and dissemination of multidrug-resistant (MDR) bacteria have largely been driven by the extensive and inappropriate use of antimicrobials in human medicine, veterinary medicine, and animal production systems, creating a complex challenge that requires coordinated interventions under the One Health framework [2,3]. Consequently, the identification of effective antimicrobial alternatives capable of reducing the selective pressure exerted by conventional antibiotics has become a global research priority.

Foods of animal origin are recognized as important reservoirs and vehicles for the dissemination of antimicrobial-resistant bacteria throughout the food chain [4]. Among these products, commercial eggs are one of the most widely consumed foods worldwide and may become contaminated during production, handling, storage, or commercialization, facilitating the transmission of potentially pathogenic bacteria to consumers [5]. Several studies have reported the isolation of species belonging to the genera *Escherichia*, *Enterobacter*, *Staphylococcus*, *Enterococcus*, *Proteus*, and *Acinetobacter* from eggs intended for human consumption, many of which exhibit multidrug-resistant phenotypes [6,7]. Beyond the direct risk posed by foodborne infections, contaminated eggs may contribute to the dissemination of antimicrobial resistance genes through the food chain, representing an important concern from a One Health perspective because they constitute a direct interface between animal production and human health [8]. In addition, *Salmonella* contamination of eggs remains a relevant food-safety concern because its occurrence may be influenced by production systems and because inadequate handling practices can facilitate household cross-contamination [9].

The increasing prevalence of MDR bacteria has markedly reduced the effectiveness of numerous antimicrobial agents used in both human and veterinary medicine [1]. This phenomenon has been associated with the widespread use and misuse of antibiotics across different production sectors, promoting the selection, persistence, and dissemination of resistant microorganisms in animals, foods, and the environment [10]. At the same time, the discovery and development of new antimicrobial agents have declined considerably during recent decades, limiting the therapeutic options available to control infections caused by resistant bacteria [11]. Therefore, there is an urgent need to identify complementary antimicrobial strategies capable of controlling resistant bacteria while reducing dependence on conventional antibiotics [12].

Among the natural alternatives currently under investigation, essential oils have attracted considerable attention because of their broad-spectrum antimicrobial activity and their ability to target multiple bacterial structures and metabolic pathways simultaneously [13]. These complex mixtures of plant secondary metabolites are mainly composed of terpenes, terpenoids, and phenolic compounds, many of which exhibit antibacterial activity against both Gram-positive and Gram-negative bacteria [14]. Unlike conventional antibiotics that generally act on a single cellular target, essential oils may simultaneously affect the bacterial cell membrane, cell wall, enzymatic systems, energy metabolism, and intracellular homeostasis, potentially reducing the likelihood of resistance development [15]. These characteristics have increased interest in their application as natural antimicrobial agents for improving food safety and reducing the dissemination of resistant bacteria within food production systems [16].

Among these natural products, the essential oil obtained from *Syzygium aromaticum* (clove) has received particular attention because of its high eugenol content, a phenolic compound widely recognized for its antibacterial, antioxidant, and anti-inflammatory properties [17,18]. Previous studies have demonstrated the antimicrobial activity of clove essential oil and eugenol against clinically relevant foodborne bacterial species, including *Escherichia*, *Staphylococcus*, *Salmonella*, *Listeria*, and *Pseudomonas* [19,20]. This activity has been associated primarily with increased membrane permeability, leakage of intracellular constituents, and disruption of essential metabolic processes required for bacterial survival [21]. In parallel, computational approaches such as molecular docking have emerged as valuable tools for predicting interactions between bioactive compounds and essential bacterial proteins, providing structural hypotheses that can complement experimental observations.

Although the antibacterial and membrane-disrupting effects of clove essential oil have been widely documented, evidence regarding its activity against taxonomically diverse multidrug-resistant bacteria associated with the food chain remains limited. Few studies have related the chemical composition of eugenol-rich clove essential oil to its bactericidal activity, sustained growth inhibition, effects on membrane permeability, and predicted interactions with bacterial proteins. Examining these effects in food-associated multidrug-resistant isolates may provide a basis for developing complementary microbial-control strategies aimed at limiting the persistence and dissemination of resistant bacteria through the food chain.

Therefore, this study aimed to evaluate the bactericidal activity of eugenol-rich *Syzygium aromaticum* essential oil against multidrug-resistant bacteria isolated from commercial eggs, assess its effects on bacterial growth and membrane integrity, and explore potential molecular interactions between eugenol and selected bacterial proteins through molecular docking.

## 2. Materials and Methods

### 2.1. Plant Material

Dried flower buds of *Syzygium aromaticum* (L.) Merr. & L.M.Perry were purchased from a local commercial supplier in Tulancingo, Hidalgo, Mexico. The material consisted of food-grade cloves intended for human consumption. Before essential oil extraction, the flower buds were manually cleaned to remove foreign material and visibly damaged tissues. Subsequently, the plant material was stored in a dry environment, protected from direct light, and maintained at room temperature until use.

### 2.2. Essential Oil Extraction

The essential oil from *Syzygium aromaticum* (SAEO) was extracted by steam distillation. Briefly, 400 g of dried flower buds was mixed with 2 L of distilled water and subjected to steam distillation for 1 h. The resulting distillate, consisting of the essential oil and hydrosol, was collected in a sterile glass container, after which the essential oil was separated from the aqueous phase using a separatory funnel.

The recovered essential oil was transferred to airtight amber glass vials and stored at 4 °C, protected from light, until chemical characterization and antimicrobial assays were performed. The extraction yield was calculated as the percentage (*v*/*w*) of essential oil recovered relative to the initial dry weight of the plant material.

### 2.3. Chemical Characterization of Syzygium aromaticum Essential Oil

The chemical composition of *Syzygium aromaticum* essential oil (SAEO) was determined by gas chromatography–mass spectrometry (GC–MS) at the National Laboratory of Macromolecular Structure of the Chemical Research Center, Autonomous University of the State of Morelos (LANEM-CIQ-UAEM, Mexico). Analyses were performed using an Agilent 7890B gas chromatograph coupled to an Agilent 7000D triple quadrupole mass spectrometer (Agilent Technologies, Santa Clara, CA, USA) operating under electron ionization (EI) mode at 70 eV.

Helium was used as the carrier gas at a constant flow rate of 1.3 mL/min. Prior to analysis, the essential oil was diluted in hexane (1:5, *v*/*v*), and 1 μL of the diluted sample was injected into the chromatographic system. Individual constituents were identified by comparing their mass spectra with those available in reference libraries and by calculating their retention indices (RI), which were determined relative to a homologous series of *n*-alkanes (C7–C30). The relative abundance of each compound was estimated from its percentage peak area in the total ion chromatogram (TIC).

### 2.4. Bacterial Strains

The bacterial isolates evaluated in this study were obtained from the culture collection of the Bacteriology Research Laboratory, Institute of Agricultural Sciences, Autonomous University of the State of Hidalgo (UAEH, Mexico). Ten bacterial isolates previously recovered from commercial eggs were selected based on their antimicrobial resistance profiles.

The Gram-positive group included *Staphylococcus xylosus*, *Staphylococcus kloosii*, *Bacillus methylotrophicus*, *Bacillus siamensis*, and *Enterococcus faecium*. The Gram-negative group comprised *Escherichia coli*, *Acinetobacter baumannii*, *Enterobacter cloacae*, *Enterobacter ludwigii*, and *Proteus mirabilis*. The complete resistance profiles and the antimicrobial agents evaluated are provided in Supplementary Tables S1 and S2.

*Staphylococcus aureus* ATCC 6538 and *Escherichia coli* ATCC 35218 were included as reference strains in the antimicrobial susceptibility, growth kinetics, and cell membrane integrity assays.

### 2.5. Determination of Minimum Inhibitory Concentration (MIC) and Minimum Bactericidal Concentration (MBC)

The minimum inhibitory concentration (MIC) and minimum bactericidal concentration (MBC) of *Syzygium aromaticum* essential oil (SAEO) were determined using the broth microdilution method according to the Clinical and Laboratory Standards Institute (CLSI) guidelines [22] and the methodology described by Zaragoza-Bastida et al. [23], with minor modifications.

Briefly, bacterial isolates were cultured in nutrient broth (DIFCO^®^) at 37 °C for 24 h, and the resulting suspensions were adjusted to a 0.5 McFarland (Remel, R20421) turbidity standard (approximately 1 × 10^8^ CFU/mL). SAEO was emulsified with 0.5% (*v*/*v*) Tween 80 and serially diluted in nutrient broth to obtain essential oil concentrations ranging from 1.0 to 0.125 mg/mL. Consequently, the Tween 80 (Tween^®^ 80) concentration decreased proportionally across the serial dilutions.

The assay was performed in sterile 96-well microplates by dispensing 90 μL of each SAEO dilution and 10 μL of the bacterial suspension into each well, resulting in a final volume of 100 μL. Microplates were incubated at 37 °C for 24 h.

Following incubation, bacterial viability was assessed by adding 20 μL of 0.04% (*w*/*v*) *p*-iodonitrotetrazolium chloride (Sigma-Aldrich 18377, St. Louis, MO, USA) solution to each well, followed by incubation for an additional 30 min. The MIC was defined as the lowest concentration of SAEO that completely inhibited visible bacterial growth, as indicated by the absence of color change from yellow to pink.

To determine the MBC, 5 μL aliquots from wells showing no visible growth were streaked onto Mueller–Hinton agar plates and incubated at 37 °C for 24 h. The MBC was defined as the lowest SAEO concentration that produced no visible bacterial colonies, corresponding to ≥99.9% bacterial killing.

The antibacterial effect of SAEO was further characterized by calculating the MBC/MIC ratio for each isolate. Ratios ≤ 4 were interpreted as indicative of bactericidal activity, whereas ratios > 4 were considered indicative of bacteriostatic activity [24].

Kanamycin (AppliChem 4K10421™, Darmstadt, Germany) was included as the positive control. Inoculated nutrient broth containing 0.5% (*v*/*v*) Tween 80 without SAEO served as the vehicle control, representing the highest surfactant concentration used in the assay. Inoculated nutrient broth without SAEO or Tween 80 and uninoculated nutrient broth served as the growth and sterility controls, respectively. Four independent biological replicates were performed on separate occasions using freshly prepared bacterial cultures and independently prepared SAEO dilutions. Within each biological replicate, each treatment and control was evaluated in four technical replicate wells. This replicate structure was applied to the bacterial growth kinetics and membrane permeability assays.

### 2.6. Growth Kinetics Assay

The effect of *Syzygium aromaticum* essential oil (SAEO) on bacterial growth over time was evaluated by monitoring growth kinetics according to the methodology described by García-Hernández et al. (2025), with minor modifications [25]. The assay included the ten multidrug-resistant bacterial isolates selected for this study and the reference strains *Staphylococcus aureus* ATCC 6538 and *Escherichia coli* ATCC 35218.

The culture medium, inoculum preparation, SAEO emulsification procedure, and experimental controls were identical to those described for MIC and MBC determination. SAEO was evaluated at 0.25 and 0.50 mg/mL, corresponding to the highest MIC and MBC values, respectively, obtained among the bacterial strains. Kanamycin (0.80 µg/mL) was included as the antibacterial control. Inoculated nutrient broth containing 0.5% (*v*/*v*) Tween 80 without SAEO served as the vehicle control, whereas inoculated nutrient broth without SAEO or Tween 80 served as the growth control. Uninoculated nutrient broth was used as sterility control.

For each treatment and control, 90 µL of the corresponding preparation was dispensed into sterile 96-well microplates, followed by the addition of 10 µL of a bacterial suspension adjusted to a 0.5 McFarland standard. The plates were incubated at 37 °C, and bacterial growth was monitored by measuring optical density at 620 nm using a Multiskan™ FC Microplate Photometer (Thermo Fisher Scientific, Vantaa, Finland).

Optical density readings were recorded every 30 min during the first 3 h of incubation and subsequently at 6, 12, and 24 h. Changes in optical density were used to characterize bacterial growth kinetics and evaluate the growth-inhibitory effect of SAEO relative to the experimental controls. Four independent biological replicates were conducted on separate occasions using freshly prepared bacterial cultures and independently prepared SAEO dilutions.

### 2.7. Cell Membrane Integrity Assay

Cell membrane permeability was evaluated using *Staphylococcus aureus* ATCC 6538 and *Escherichia coli* ATCC 35218 as representative Gram-positive and Gram-negative bacteria, respectively. The assay was performed according to the methodology described by García-Hernández et al. [25], with minor modifications.

Bacterial suspensions were exposed to SAEO at concentrations equivalent to the MBC and 2× MBC and incubated at 37 °C for 30 min. Sterile saline solution was used as the negative control, whereas a cell lysis solution (Wizard^®^ Genomic DNA Purification Kit, Promega, Madison, WI, USA) and thermal shock treatment (96 °C for 10 min followed by 20 °C for 5 min) were included as positive controls.

Following treatment, the bacterial suspensions were centrifuged at 10,000 rpm for 5 min at 4 °C, and the cell-free supernatants were collected. Extracellular nucleic acids were estimated from absorbance at 260 nm using the nucleic acid module of a NanoDrop™ 1000 spectrophotometer (Thermo Fisher Scientific, Wilmington, DE, USA) and the instrument’s conversion factor for double-stranded DNA. Extracellular protein was estimated from absorbance at 280 nm using the Protein A280 module. No external standard curves were constructed; therefore, the instrument-generated values were considered semiquantitative estimates rather than absolute concentrations. Increased values relative to the negative control were interpreted as indirect evidence of extracellular release of UV-absorbing cellular material and altered membrane permeability.

### 2.8. In Silico Molecular Docking Analysis

#### 2.8.1. Preparation of Eugenol

To obtain a suitable structural representation of eugenol for molecular docking studies, a conformational search was initially performed using semi-empirical methods. A set of conformers corresponding to local minima on the potential energy surface was generated using the AM1 method in Spartan 06 [26]. Subsequently, the lowest-energy conformers were selected for geometric optimization using Density Functional Theory (DFT). The optimized structure and minimum energy of the compound were determined using the B3LYP hybrid functional with the 6-311++G(d,p) basis set, a methodology widely used for computational studies of bioactive organic compounds [27,28]. The quantum-mechanical calculations for the molecule’s ground state were performed using Gaussian 16 [29], while molecular construction, visualization, and analysis of the optimized geometry were carried out using GaussView 6.0 [30]. The optimized molecular geometry was subsequently used as the input ligand structure for the molecular docking studies.

#### 2.8.2. Enzyme Preparation

The crystallographic structures of dihydropteroate synthase (DHPS) from *Escherichia coli* and β-ketoacyl-ACP synthase III (FabH) from *Staphylococcus aureus* were obtained from the Protein Data Bank (PDB) using the accession codes 1AJ0 and 3IL7, respectively [31,32]. Both enzymes were selected due to their essential role in bacterial metabolic processes. DHPS participates in folate biosynthesis through the condensation of *p*-aminobenzoic acid (PABA) with 6-hydroxymethyl-7,8-dihydropterin pyrophosphate, an indispensable reaction for nucleotide production and bacterial DNA synthesis [33]. On the other hand, FabH catalyzes the initial condensation reaction within the type II fatty acid synthesis (FAS-II) pathway, considered one of the rate-limiting steps for the formation of membrane lipids in bacteria [34].

Prior to molecular docking studies, the protein structures were prepared by removing water molecules, cofactors, and crystallographic ligands present in the original files. Subsequently, polar hydrogens were added, and partial charges were assigned using the tools available in AutoDock Tools 1.5.7 [35,36]. The definition of the binding sites was performed using a grid-box-based procedure centered on the catalytic regions of each protein. For FabH (PDB ID: 3IL7), a rectangular grid of 55 × 115 × 80 Å was used, while for DHPS (PDB ID: 1AJ0), a grid of 60 × 70 × 80 Å was employed, with a spacing of 0.375 Å between grid points. For FabH, the region corresponding to the catalytic triad responsible for the acyl condensation reaction was considered. In the structure used, this region is composed of residues Cys112, His238, and Asn268, equivalent to the conserved catalytic residues described for other bacterial FabH enzymes [37]. In turn, the active site of DHPS is constituted by conserved residues involved in substrate recognition and the catalytic process, among which Asn22, Thr62, Arg63, Asp96, Asn115, Ile117, Asp185, Phe190, and His257 stand out [38].

To establish a reference for evaluating the affinity of eugenol, molecular docking studies were performed using sulfamethoxazole and thiolactomycin as reference ligands for DHPS and FabH, respectively. Both compounds have been widely reported as inhibitors of these enzymes and were used as positive controls to compare the binding energies and interaction modes obtained for eugenol [36,37].

#### 2.8.3. Molecular Docking

Molecular docking studies were performed using the AutoDock 4.2 program, which enables prediction of possible binding orientations between a ligand and a biological macromolecule through search algorithms and energy scoring functions [33,38]. All simulations were executed using the hybrid Lamarckian genetic algorithm, with an initial population of 100 randomly distributed individuals and a maximum of 1.0 × 10^7^ energy evaluations. The remaining parameters were kept at their default settings. For each protein–ligand system, multiple binding poses were generated and ranked according to their predicted binding free energy (ΔG). The pose with the most favorable predicted ΔG value was selected for subsequent structural analysis. Likewise, the residues involved in the protein–ligand interaction and the types of interaction present in each complex were identified, including hydrogen bonds, hydrophobic interactions, and aromatic contacts, to characterize the predicted binding mode of eugenol within each target and compare its predicted binding energy and molecular interactions with those of the corresponding reference ligand.

### 2.9. Experimental Design and Statistical Analysis

Minimum inhibitory concentration (MIC) and minimum bactericidal concentration (MBC) values were transformed using base-10 logarithms (log10) to satisfy the assumptions of normality and homogeneity of variance required for parametric analyses. Data was analyzed using a completely randomized design under a General Linear Model (GLM).

Differences among treatment means were determined using Tukey’s multiple comparison test, considering a significance level of α = 0.05. Statistical analyses were performed using SAS software version 9.0 (SAS Institute Inc., Cary, NC, USA). Each well was considered an independent experimental unit.

## 3. Results

### 3.1. Chemical Composition of Syzygium aromaticum Essential Oil

Steam distillation of *Syzygium aromaticum* flower buds yielded 2.5% (*v*/*w*) essential oil. GC–MS analysis tentatively identified five major compounds above the 1% reporting threshold (Table 1). Minor or unresolved signals were excluded before calculating the relative composition. The peak areas of the five reported compounds were normalized to their summed area and expressed as relative percentages. Eugenol was the predominant constituent (73.9%), followed by dihydroeugenyl acetate (14.1%), β-caryophyllene (6.0%), chavibetol (m-eugenol; 4.2%), and 3-allylguaiacol (1.8%).

### 3.2. Antibacterial Activity of Syzygium aromaticum Essential Oil

*Syzygium aromaticum* essential oil (SAEO) exhibited antibacterial activity against all bacterial strains evaluated, with minimum inhibitory concentration (MIC) values ranging from 0.12 to 0.25 mg/mL (Table 2). The lowest MIC value (0.12 mg/mL) was observed in seven of the twelve bacterial strains evaluated, including *Staphylococcus xylosus*, *Staphylococcus kloosii*, *Bacillus siamensis*, *Acinetobacter baumannii*, *Enterobacter cloacae*, *Enterobacter ludwigii*, and *Proteus mirabilis*, whereas the remaining strains exhibited MIC values of 0.25 mg/mL. Statistical analysis revealed significant differences among bacterial species (*p* ≤ 0.05). However, when strains were grouped according to cell wall structure, no significant differences were observed between Gram-positive (0.20 mg/mL) and Gram-negative bacteria (0.15 mg/mL) (*p* = 0.242).

Minimum bactericidal concentration (MBC) values ranged from 0.25 to 0.50 mg/mL. The lowest MBC values (0.25 mg/mL) were observed for *Staphylococcus xylosus*, *Staphylococcus kloosii*, *Bacillus siamensis*, *Enterobacter cloacae*, and *Enterobacter ludwigii*, whereas the remaining strains required 0.50 mg/mL to achieve a bactericidal effect. Although significant differences were detected among bacterial species (*p* ≤ 0.05), the mean MBC values were identical for Gram-positive and Gram-negative bacteria (0.40 mg/mL).

The MBC/MIC ratio ranged from 2 to 4 for all strains tested, indicating bactericidal activity against 100% of the evaluated microorganisms. Overall, these findings demonstrate a consistent antibacterial effect of SAEO regardless of the Gram classification of the bacterial strains (Table 2).

### 3.3. Bacterial Growth Curves

**Figure 1:**
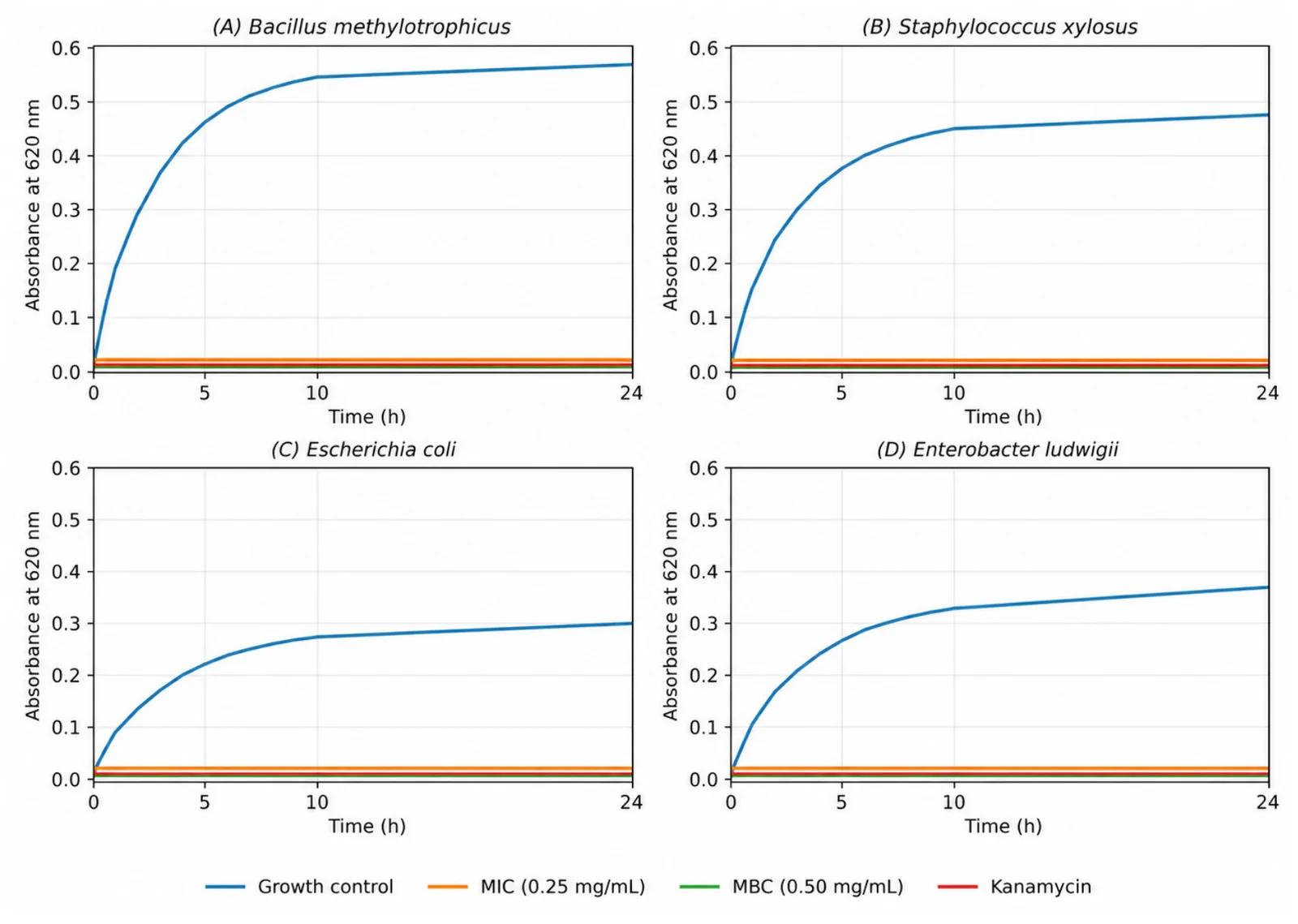
Representative growth curves of Gram-positive and Gram-negative bacteria exposed to *Syzygium aromaticum* essential oil (SAEO). (**A**) *Bacillus methylotrophicus*; (**B**) *Staphylococcus xylosus*; (**C**) *Escherichia coli*; and (**D**) *Enterobacter ludwigii*. Bacterial growth was monitored by measuring optical density at 620 nm over a 24-h incubation period. SAEO was evaluated at MIC (0.25 mg/mL) and MBC (0.50 mg/mL) concentrations. Kanamycin was included as a positive control. Growth controls consisted of untreated bacterial cultures.

The growth curves showed a progressive increase in optical density in untreated cultures throughout the 24-h incubation period (Figure 1). In contrast, exposure to *Syzygium aromaticum* essential oil at MIC (0.25 mg/mL) and MBC (0.50 mg/mL) concentrations maintained absorbance values close to those observed for kanamycin-treated cultures, indicating effective inhibition of bacterial growth. This inhibitory effect was consistently observed in both Gram-positive and Gram-negative bacteria.

### 3.4. Cell Membrane Integrity

**Figure 2:**
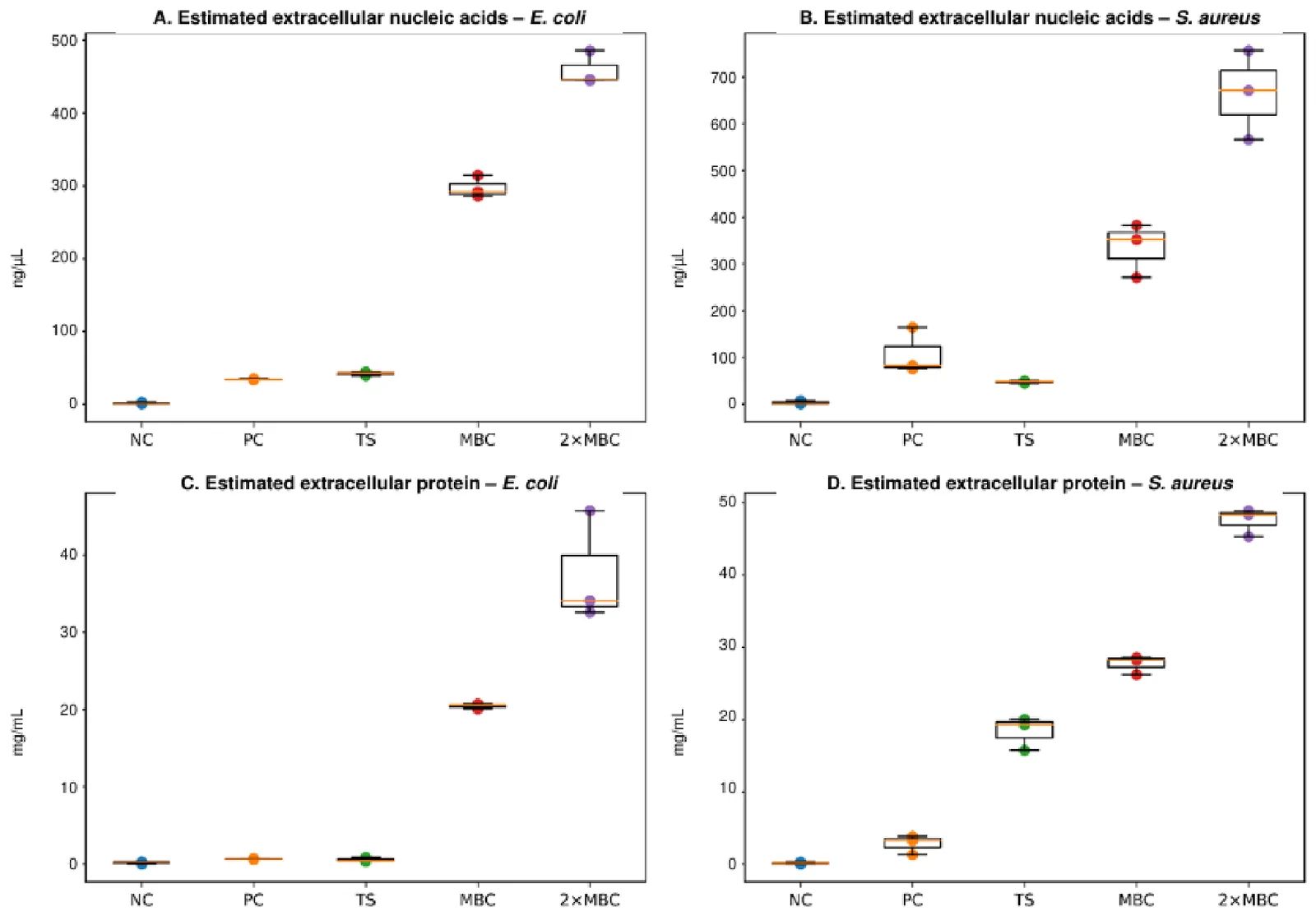
Effect of *Syzygium aromaticum* essential oil (SAEO) on the estimated extracellular release of nucleic acids and proteins by *Escherichia coli* and *Staphylococcus aureus*. Estimated extracellular nucleic acid concentrations in *E. coli* (**A**) and *S. aureus* (**B**) and estimated extracellular protein concentrations in *E. coli* (**C**) and *S. aureus* (**D**). Bacterial suspensions were exposed to SAEO at the minimum bactericidal concentration (MBC) and twice the MBC (2× MBC) for 30 min. Sterile saline solution was used as the negative control (NC), whereas cell lysis solution (PC) and thermal shock treatment (TS) were included as positive controls. Nucleic acid and protein values are expressed as ng/µL and mg/mL, respectively, and represent semiquantitative estimates generated from absorbance measurements at 260 and 280 nm. Significant differences were detected among treatments and between bacterial species (*p* < 0.05).

Exposure to SAEO significantly increased the absorbance of cell-free supernatants at 260 and 280 nm in both Escherichia coli and Staphylococcus aureus compared with the negative control (Figure 2). At both wavelengths, the highest absorbance values among the SAEO treatments were observed at 2× MBC, followed by MBC, whereas the lowest values were recorded in the untreated control. Staphylococcus aureus exhibited significantly higher absorbance values than E. coli under the same experimental conditions (*p* < 0.05). Significant differences were also detected among SAEO treatments (*p* < 0.05), with absorbance increasing at the higher SAEO concentration. These findings are consistent with increased extracellular release of UV-absorbing cellular material and provide indirect evidence of concentration-associated alterations in bacterial membrane permeability.

### 3.5. Optimized Structure of Eugenol

**Figure 3:**
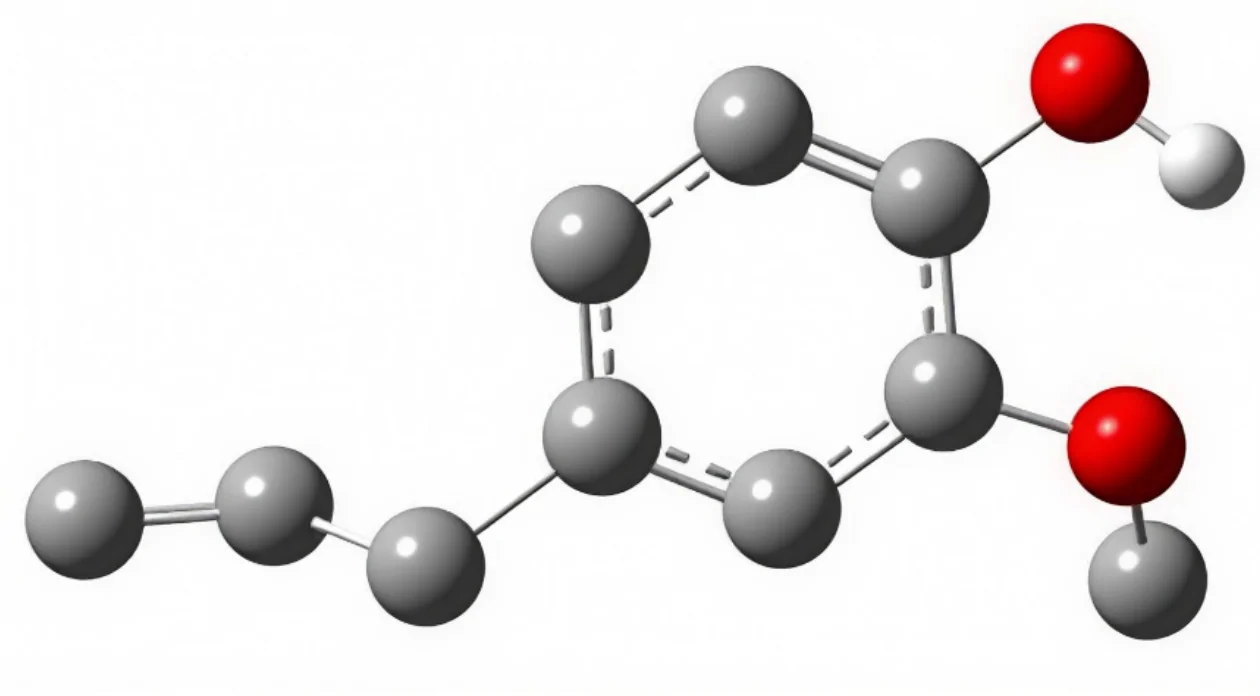
Eugenol—optimized molecule. Gray, red, and white spheres represent carbon, oxygen, and hydrogen atoms, respectively. Dashed lines within the aromatic ring represent delocalized aromatic π bonds.

The optimized molecule was eugenol, shown in Figure 3.

**Figure 4:**
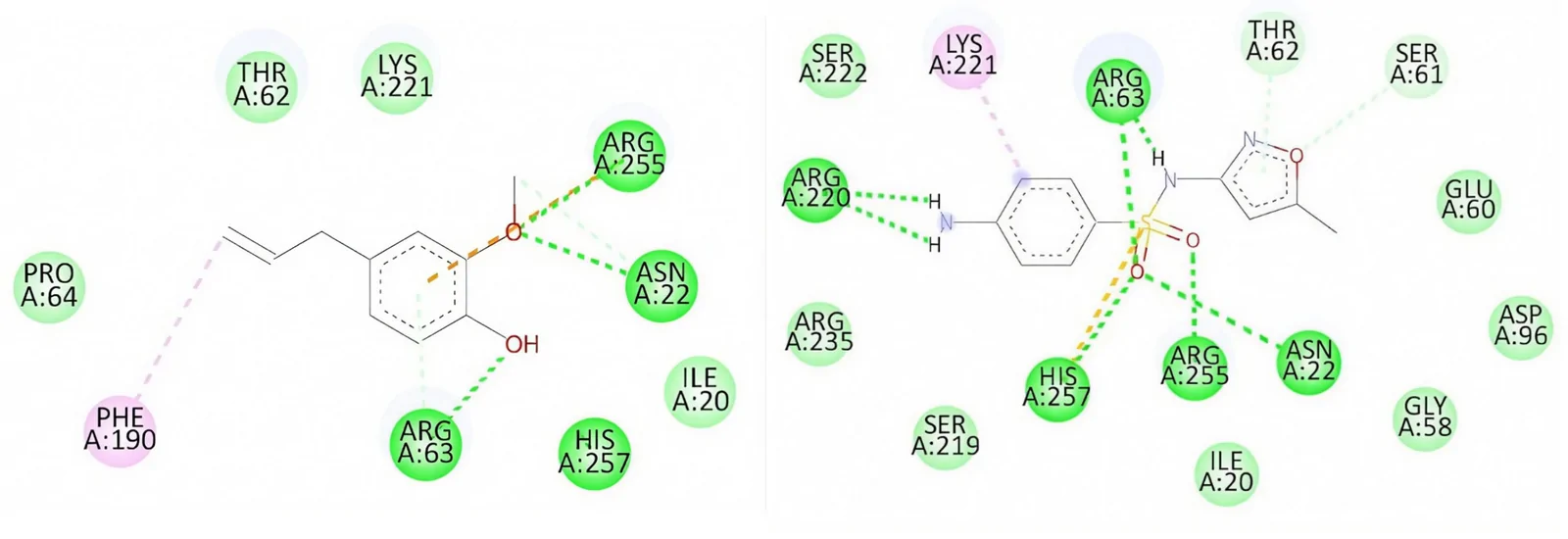
Interaction map of eugenol (**left**) and sulfamethoxazole (**right**) in the active site of DHPS. Green dashed lines indicate conventional hydrogen bonds; light-green dashed lines indicate carbon–hydrogen or π-donor hydrogen-bond interactions; orange and yellow dashed lines indicate π–cation and π–sulfur interactions, respectively; and pink dashed lines indicate hydrophobic π–alkyl interactions. Residues displayed in pale green represent van der Waals contacts. Detailed interactions are presented in Table 3.

**Figure 5:**
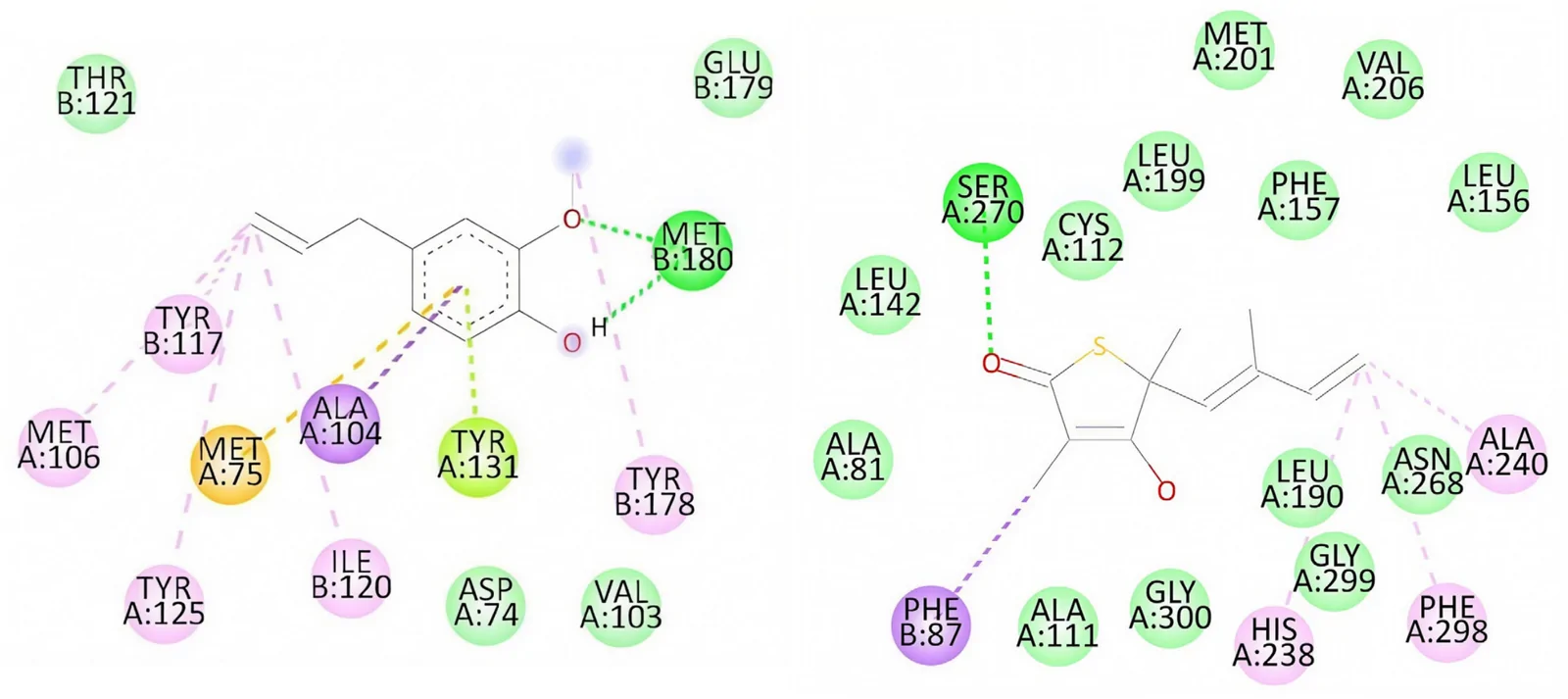
Interaction map of eugenol (**left**) and thiolactomycin (**right**) in the active site of FabH. Green dashed lines indicate conventional hydrogen bonds; purple, yellow, and light-green dashed lines indicate π–sigma, π–sulfur, and π–lone pair interactions, respectively; and pink dashed lines indicate aromatic or hydrophobic interactions, including T-shaped π–π, alkyl, and π–alkyl contacts. Residues displayed in pale green represent van der Waals contacts. Detailed interactions are presented in Table 3.

Molecular docking predicted that eugenol could bind to both protein targets; however, its binding free energy (ΔG) values were less favorable than those of the corresponding reference ligands. For DHPS, the predicted ΔG values were −4.86 kcal/mol for eugenol and −7.45 kcal/mol for sulfamethoxazole (Figure 4). For FabH, the predicted ΔG values were −5.18 kcal/mol for eugenol (Figure 5) and −6.48 kcal/mol for thiolactomycin. The predicted binding energies are summarized in Table 3.

In the predicted DHPS–eugenol binding pose, conventional hydrogen bonds were identified with Asn22, Arg63, and Arg255, along with a carbon–hydrogen bond with Asn22. A π–cation interaction with Arg255 and a hydrophobic π–alkyl interaction with Phe190 were also predicted. Additional van der Waals contacts involved Ile20, Thr62, Pro64, Lys221, and His257. These interactions may contribute to the stabilization of the modeled protein–ligand complex (Figure 4).

The predicted DHPS–sulfamethoxazole binding pose showed conventional hydrogen bonds with Asn22, Arg63, Arg220, Arg255, and His257, as well as π-donor hydrogen-bond interactions with Ser61 and Thr62. A π–sulfur interaction with His257 and a π–alkyl interaction with Lys221 were also identified. Additional van der Waals contacts involved Ile20, Gly58, Glu60, Asp96, Ser219, Ser222, and Arg235 (Figure 4).

In the FabH–eugenol complex, the formation of a conventional hydrogen bond with Met180 was observed. Additionally, π-sigma interactions with Ala104, a π–sulfur interaction with Met75, a π-lone pair interaction with Tyr131, and a T-shaped π–π aromatic interaction with Tyr178 were identified. Likewise, hydrophobic π–alkyl interactions with Met106, Tyr117, Ile120, and Tyr125 were detected, in addition to van der Waals contacts with Asp74, Val103, Thr121, and Glu179 (Figure 5).

Finally, thiolactomycin exhibited the highest affinity for FabH, with a binding free energy of −6.48 kcal/mol. This complex showed a conventional hydrogen bond with Ser270, a π-sigma interaction with Phe87, and alkyl and π–alkyl interactions with His238, Ala240, and Phe298. Furthermore, numerous van der Waals contacts were identified with Ala81, Ala111, Cys112, Leu142, Leu156, Phe157, Leu190, Leu199, Met201, Val206, Asn268, Gly299, and Gly300 (Figure 5).

## 4. Discussion

GC–MS analysis revealed that eugenol was the predominant constituent of *Syzygium aromaticum* essential oil, accounting for 73.9% of the total composition, followed by dihydroeugenyl acetate (14.1%) and β-caryophyllene (6.0%). This composition is consistent with previous reports describing clove essential oils obtained from flower buds, in which eugenol typically represents between 60% and 85% of the volatile fraction and is recognized as the primary contributor to the biological properties of this essential oil [17,18]. Variations in the relative abundance of chemical constituents may be influenced by several factors, including geographical origin, cultivation conditions, plant physiological status, and extraction methodology [18]. Due to its phenolic and hydrophobic nature, eugenol has been widely associated with the antibacterial activity of clove essential oil because of its ability to interact with bacterial cellular structures and disrupt essential physiological processes required for microbial survival [19]. Therefore, the high proportion of eugenol identified in the present study contributed to the antibacterial activity observed against the multidrug-resistant bacteria evaluated.

*Syzygium aromaticum* essential oil exhibited antibacterial activity against all bacterial strains evaluated, with MIC values ranging from 0.12 to 0.25 mg/mL and MBC values ranging from 0.25 to 0.50 mg/mL. These findings demonstrate the high susceptibility of multidrug-resistant bacteria isolated from commercial eggs and are consistent with previous studies reporting significant antimicrobial activity of clove essential oil against clinically and food-relevant bacterial species [19,20]. The MIC and MBC values obtained in the present study fall within the range previously reported for bacterial species belonging to the genera *Escherichia*, *Staphylococcus*, *Enterococcus*, and *Acinetobacter*, further supporting the broad-spectrum antibacterial activity attributed to this essential oil [20,21]. Collectively, these findings support the potential of *S. aromaticum* essential oil as a source of antibacterial compounds active against multidrug-resistant bacteria associated with foods of avian origin.

A relevant finding of this study was the absence of significant differences in the antibacterial activity of *S. aromaticum* essential oil between Gram-positive and Gram-negative bacteria. Gram-negative bacteria are generally considered more tolerant to antimicrobial agents due to the presence of an outer membrane rich in lipopolysaccharides that acts as an effective permeability barrier against exogenous compounds [39]. However, the MIC and MBC values obtained in the present study did not differ significantly between bacterial groups, suggesting that the bioactive constituents present in the essential oil were able to exert antibacterial activity regardless of bacterial cell wall structure. Consistent with these findings, previous studies have reported similar results for eugenol-rich essential oils, in which the hydrophobic nature of eugenol facilitates its interaction with biological membranes and promotes antibacterial activity against microorganisms belonging to different taxonomic groups [15,19]. These results suggest that *S. aromaticum* essential oil possesses broad-spectrum antibacterial activity, a characteristic of relevance for controlling multidrug-resistant bacteria associated with foods.

The MBC/MIC ratio is one of the most widely accepted criteria for distinguishing between bacteriostatic and bactericidal effects of antimicrobial agents. In the present study, MBC/MIC ratios ranged from 2 to 4 for all bacterial strains evaluated, indicating bactericidal activity against 100% of the tested microorganisms. According to the criteria proposed for natural products, MBC/MIC ratios ≤ 4 indicate bactericidal activity, whereas values > 4 suggest a predominantly bacteriostatic effect [40]. These results demonstrate that *S. aromaticum* essential oil not only inhibited bacterial growth but was also capable of eliminating viable bacterial cells, even in strains exhibiting multidrug-resistant phenotypes. Similar findings have been reported for eugenol-rich essential oils, where bactericidal activity has been associated with irreversible damage to essential cellular structures and disruption of bacterial homeostasis [19,29]. The bactericidal activity observed in this study further supports the potential use of clove essential oil as a natural alternative for controlling multidrug-resistant bacteria associated with foods.

The bacterial growth curves supported the antibacterial activity observed in the broth microdilution assays. Both Gram-positive and Gram-negative bacteria exposed to *S. aromaticum* essential oil at the evaluated MBC exhibited sustained growth inhibition throughout the 24-h incubation period, with optical density values like those recorded for kanamycin-treated cultures. In contrast, the untreated controls showed a progressive increase in optical density. These findings indicate that the inhibitory effect of SAEO was maintained throughout the experimental period, limiting bacterial proliferation without a detectable rebound in growth. Similar findings have been reported for eugenol-rich essential oils, which produce sustained inhibition of bacterial growth through cellular alterations affecting essential physiological functions [41,42].

The membrane permeability assay provided indirect evidence that SAEO altered the bacterial cell envelope. Exposure of *Escherichia coli* and *Staphylococcus aureus* to SAEO produced higher estimated extracellular nucleic acid and protein values, particularly at 2× MBC. The increased detection of UV-absorbing cellular material in cell-free supernatants is consistent with increased membrane permeability and extracellular leakage of intracellular components [21,43]. A concentration-associated response was observed, with the highest estimates recorded at 2× MBC. Comparable alterations in membrane permeability and leakage of intracellular material have been reported following exposure to eugenol and eugenol-rich essential oils [19,29,31]. These spectrophotometric measurements provide indirect and semiquantitative evidence of membrane alteration and do not identify the released molecules or establish a specific molecular mechanism.

Molecular docking was used to examine the predicted binding of eugenol to DHPS and FabH. The binding energies were −4.86 kcal/mol for DHPS and −5.18 kcal/mol for FabH, with hydrogen-bonding and hydrophobic contacts detected within their binding regions. The reference ligands were included only as controls for the docking procedure because their interactions with the respective enzymes have been previously characterized [36,37]. The results do not demonstrate inhibition of either enzyme or show that these proteins are responsible for the antibacterial activity recorded in vitro. Enzyme-inhibition assays are required to determine whether the predicted binding has a functional effect.

Eugenol was the predominant constituent of the essential oil (73.9%), but compounds detected at lower relative abundances may also contribute to its antibacterial activity. Chavibetol represented 4.2% of the composition and has a chemical structure similar to that of eugenol. It may act individually or in combination with other constituents. However, chavibetol was not tested separately or included in the docking analysis; therefore, its contribution to the antibacterial activity cannot be determined from the present results. The docking analysis was limited to eugenol and should not be interpreted as evidence that this compound alone accounts for the activity of the essential oil.

The detection of multidrug-resistant bacteria in commercial eggs is relevant to food safety because resistant microorganisms can enter the food chain. The activity of *S. aromaticum* essential oil against these isolates supports further investigation of its use as part of microbial-control measures during egg production, handling, or processing. However, the experiments were conducted under control in vitro conditions, and the results do not demonstrate that the essential oil is effective when applied directly to eggs or other food matrices.

Further studies should determine the effective concentration, stability, safety, sensory effects, and compatibility of *S. aromaticum* essential oil with food products and food-contact materials. Its performance should also be evaluated together with established hygiene and biosecurity measures under production and processing conditions. At this stage, the essential oil should be considered a candidate for food-safety applications rather than a substitute for conventional antimicrobial therapy.

## 5. Conclusions

The essential oil of *Syzygium aromaticum*, characterized by a high eugenol content (73.9%), exhibited antibacterial activity against all multidrug-resistant bacterial isolates evaluated, with MIC values ranging from 0.12 to 0.25 mg/mL and MBC/MIC ratios ≤ 4, indicating a bactericidal effect. Comparable activity against Gram-positive and Gram-negative bacteria supported its broad antibacterial spectrum. The essential oil also inhibited bacterial growth for up to 24 h and promoted the extracellular release of nucleic acids and proteins, findings consistent with loss of cell membrane integrity. Molecular docking suggested plausible interactions between eugenol and DHPS and FabH; however, these predictions do not confirm enzyme inhibition or establish a specific mechanism of action. Overall, the findings support the potential of *S. aromaticum* essential oil as a natural antibacterial agent against multidrug-resistant bacteria associated with commercial eggs. Further studies should validate the predicted molecular targets and evaluate their safety, efficacy, sensory effects, and technological feasibility in food production and processing systems.

## Figures and Tables

**Table 1:** Chemical constituents identified in *Syzygium aromaticum* essential oil by GC–MS analysis.

No	Compound	Molecular Formula	Retention Time (Rt, min)	Abundance (%)	Compound Type
1	Chavibetol (m-Eugenol)	C_10_H_12_O_2_	12.26	4.2	Phenylpropanoid
2	Eugenol	C_10_H_12_O_2_	12.31	73.9	Phenylpropanoid
3	β-Caryophyllene	C_15_H_24_	13.11	6.0	Sesquiterpene
4	3-allylguaiacol	C_10_H_12_O_2_	13.56	1.8	Phenylpropanoid
5	Dihydroeugenyl acetate	C_12_H_16_O_3_	15.18	14.1	Oxygenated phenylpropanoid

Rt, retention time. Compounds were tentatively identified by comparison of their mass spectra with the NIST mass spectral library. Signals below the 1% reporting threshold, unresolved peaks, and signals with insufficient spectral similarity were excluded. Relative abundances were calculated by normalizing the peak area of each reported compound to the summed peak areas of the five included compounds.

**Table 2:** Minimum inhibitory concentration (MIC), minimum bactericidal concentration (MBC), and MBC/MIC ratio of *Syzygium aromaticum* essential oil (SAEO) against multidrug-resistant bacteria isolated from commercial eggs and reference strains.

Bacterial Strain	MIC (mg/mL) SAEO	MIC (µg/mL) Kanamycin	MBC (mg/mL) SAEO	MBC (µg/mL) Kanamycin	MBC/MIC
*S. aureus* ATCC 6538	0.25 ^b^	0.10 ^c^	0.50 ^b^	0.20 ^b^	2
*S. xylosus*	0.12 ^a^	0.05 ^b^	0.25 ^a^	0.10 ^a^	2
*S. kloosii*	0.12 ^a^	0.10 ^c^	0.25 ^a^	0.40 ^c^	2
*B. methylotrophicus*	0.25 ^b^	0.40 ^d^	0.50 ^b^	0.80 ^d^	2
*B. siamensis*	0.12 ^a^	0.10 ^c^	0.25 ^a^	0.20 ^b^	2
*E. faecium*	0.25 ^b^	0.10 ^c^	0.50 ^b^	0.20 ^b^	2
*E. coli* ATCC 35218	0.25 ^b^	0.10 ^c^	0.50 ^b^	0.20 ^b^	2
*E. coli*	0.25 ^b^	0.10 ^c^	0.50 ^b^	0.20 ^b^	2
*A. baumannii*	0.12 ^a^	0.10 ^c^	0.50 ^b^	0.20 ^b^	4
*E. cloacae*	0.12 ^a^	0.05 ^b^	0.25 ^a^	0.10 ^a^	2
*E. ludwigii*	0.12 ^a^	0.01 ^a^	0.25 ^a^	0.02 ^a^	2
*P. mirabilis*	0.12 ^a^	0.05 ^b^	0.50 ^b^	0.20 ^b^	4

SAEO: *Syzygium aromaticum* essential oil. Values are expressed as mg/mL for SAEO and µg/mL for kanamycin. Different superscript letters within the same column indicate significant differences according to Tukey’s multiple comparison test (*p* ≤ 0.05).

**Table 3:** ΔG and interaction types of the two enzymes with eugenol and reference ligand.

Complex	ΔG (kcal/mol)	Residue(s)	Type of Interaction
DHPS–Eugenol	−4.86	Asn22, Arg63, Arg255	Hydrogen bridge
		Asn22	Carbon–hydrogen bridge
		Phe190	π–alkyl
		Arg255	π–cation
		Ile20, Thr62, Pro64, Lys221, His257	Van der Waals
DHPS–Sulfametoxazol	−7.45	Asn22, Arg63, Arg220, Arg255, His257	Hydrogen bridge
		Ser61, Thr62	π-donor hydrogen bond
		His257	π–sulfur
		Lys221	π–alkyl
		Ile20, Gly58, Glu60, Asp96, Ser219, Ser222, Arg235	Van der Waals
		Met180	Hydrogen bridge

## Data Availability

The datasets generated and/or analyzed during the current study are available from the corresponding author upon reasonable request. Public access to these data is restricted because they are protected by intellectual property rights.

## Supplementary Materials

The following supporting information can be downloaded at: https://www.mdpi.com/article/10.3390/pathogens15090910/s1, Table S1: Antimicrobial resistance profiles of Gram-positive bacterial isolates recovered from commercial eggs; Table S2: Antimicrobial resistance profiles of Gram-negative bacterial isolates recovered from commercial eggs.
