# Supplementary material for: Bactericidal Activity of Eugenol-Rich Syzygium aromaticum Essential Oil Against Multidrug-Resistant Bacteria Isolated from Commercial Eggs
Source: Pathogens. 2026 Aug 29;15(9):910. doi: 10.3390/pathogens15090910 (PMC13610623; doi:10.3390/pathogens15090910)
Supplement: Supplementary file 1 [file pathogens-15-00910-s001.zip › Supplementary Table S1.pdf]

Table S1. Antimicrobial resistance profiles of Gram-positive bacterial isolates recovered from commercial eggs.

Bacteria	AM	CF	CFX	DC	CPF	GE	CLM	E	STX	PE	VA	TE	MARI
Staphylococcus xylosus	R	R	R	R	R	R	R	R	R	S	R	R	0.91
Staphylococcus kloosii	R	R	R	R	R	R	R	R	S	R	R	R	0.91
Bacillus methylotrophicus	R	R	R	R	R	R	R	R	R	R	R	R	1.0
Bacillus siamensis	R	R	R	R	S	R	R	R	R	R	R	R	0.91
Enterococcus faecium	R	R	R	R	R	R	R	R	R	R	R	S	0.91

AM, ampicillin; CF, cephalothin; CFX, cefotaxime; DC, dicloxacillin; CPF, ciprofloxacin; GE, gentamicin; CLM, clindamycin; E, erythromycin; STX, trimethoprim–sulfamethoxazole; PE, penicillin; VA, vancomycin; TE, tetracycline; MARI, multiple antimicrobial resistance index; S, susceptible; R, resistant.
