# Supplementary material for: Bactericidal Activity of Eugenol-Rich Syzygium aromaticum Essential Oil Against Multidrug-Resistant Bacteria Isolated from Commercial Eggs
Source: Pathogens. 2026 Aug 29;15(9):910. doi: 10.3390/pathogens15090910 (PMC13610623; doi:10.3390/pathogens15090910)
Supplement: Supplementary file 1 [file pathogens-15-00910-s001.zip › Supplementary Table S2.pdf]

Table S2. Antimicrobial resistance profiles of Gram-negative bacterial isolates recovered from commercial eggs.

Bacteria	AM	AK	CB	GE	CF	NET	CFX	CPF	NOF	CL	STX	NF	MARI
Escherichia coli	R	S	R	S	R	R	R	R	R	R	R	R	0.83
Acinetobacter baumannii	R	R	R	S	R	R	R	S	S	R	S	R	0.67
Enterobacter cloacae	R	R	R	R	R	R	R	S	S	R	R	R	0.83
Enterobacter ludwigii	R	R	R	R	R	R	R	R	S	S	R	S	0.75
Proteus mirabilis	R	R	R	R	R	S	R	R	S	S	R	R	0.75

AM, ampicillin; CF, cephalothin; CFX, cefotaxime; DC, dicloxacillin; CPF, ciprofloxacin; GE, gentamicin; CLM, clindamycin; E, erythromycin; STX, trimethoprim–sulfamethoxazole; PE, penicillin; VA, vancomycin; TE, tetracycline; MARI, multiple antimicrobial resistance index; S, susceptible; R, resistant.
